# Clinical risk factors of carbohydrate antigen-125, cytokeratin fragment 19, and neuron-specific enolase in liver metastases from elderly lung cancer patients

**DOI:** 10.3389/fgene.2022.1013253

**Published:** 2022-09-29

**Authors:** Tao Cheng, Jun Chen, Ping Ying, Hong Wei, Huiye Shu, Min Kang, Jie Zou, Qian Ling, Xulin Liao, Yixin Wang, Yi Shao

**Affiliations:** ^1^ Department of Respiratory, Shangrao People’s Hospital of Nanchang University, Shangrao, Jiangxi, China; ^2^ Department of Ophthalmology, Jiangxi Branch of National Clinical Research Center for Ocular Disease, The First Affiliated Hospital of Nanchang University, Nanchang, Jiangxi, China; ^3^ Department of Ophthalmology and Visual Sciences, The Chinese University of Hong Kong, Hong Kong, Hong Kong SAR, China; ^4^ School of Optometry and Vision Science, Cardiff University, Cardiff, United Kingdom

**Keywords:** lung cancer, liver metastasis, risk factors, potential indicators, CA-125, CYFRA21-1, NSE

## Abstract

**Objective:** Lung cancer is a common malignant tumor characterized by challenging detection and lack of specificity in clinical manifestations. To investigate the correlation of tumor markers in the serum with liver metastasis and prognosis of lung cancer.

**Methods:** A total of 3,046 elderly lung cancer patients were retrospectively studied between September 1999 and July 2020. Divided into liver metastasis group and non-liver metastasis group. We compared a series of serum biomarkers between the two groups of elderly patients to predict the prognosis in patients with lung cancer by fluorescence *in situ* hybridization (FISH), advanced flow cytometry (FCM) and multi tumor marker protein chip, including tumor markers in the serum included alkaline phosphatase (ALP), serum calcium, hemoglobin (HB), alpha-fetoprotein (AFP), carcinoembryonic antigen (CEA), neuron-specific enolase (NSE), cytokeratin fragment 19 (Cyfra21-1), carbohydrate antigen-125 (CA-125), carbohydrate antigen-153 (CA-153), carbohydrate antigen-199 (CA-199), and free prostate specific antigen (free PSA). We used binary logistic regression analysis to determine risk factors, and used receiver operating curve (ROC) analysis to evaluate the diagnostic value of liver metastases in elderly patients with lung cancer.

**Results:** The proportion of lung cancer in the liver metastasis group was higher than that observed in the non-liver metastases group. The expression levels of CA-125, Cyfra21-1, and NSE in the liver metastasis group of lung cancer were significantly higher than those reported in the non-liver metastases group (*p* < 0.05). ROC curve analysis shows that the area under the curve of CA-125, Cyfra21-1, and NSE are 0.614, 0.616 and 0.608, respectively. The sensitivity and specificity of CA-125 were 45.70% and 76.20%, the sensitivity and specificity of Cyfra21-1 were 60.10% and 57.10%, and the sensitivity and specificity of NSE were 44.10% and 75.00%, respectively.

**Conclusion:** High levels of CA-125, Cyfra21-1, and NSE in the serum may be associated with liver metastasis in elderly patients with lung cancer. CA-125 and NSE are factors influencing the prognosis of elderly patients with liver metastasis of lung cancer.

## Introduction

The morbidity and mortality of lung cancer remain high. This type of cancer has become one of the most important life-threatening diseases in humans. According to studies, the incidence and mortality of lung cancer rank first among all types of cancer in men, accounting for 17% of the total number of new cancer cases and 23% of the total number of cancer-related deaths. (Jemal et al., 2011) Following the rapid development of medical care witnessed in recent years, the treatment of various diseases has been very successful. However, the prognosis of elderly lung cancer patients has not been effectively improved. The reason for this may be that lung cancer is a very complicated tumor, and characterized by the occurrence of metastasis. In addition, it is the main cause of treatment failure and patient death. Liver metastasis of lung cancer is one of the most common sites of hematogenous metastasis of lung cancer, and It is one of the third most common sites of liver metastasis the disease progresses rapidly and the prognosis is poor. In all elderly patients with lung cancer metastasis, the incidence of liver metastasis and adrenal metastasis was 33–40% and 18–38%, respectively. (Quint et al., 1996) Elderly patients with liver metastasis of lung cancer may not present obvious symptoms in the early stage. Thus, the diagnosis mainly depends on imaging. Computed tomography (CT) is an accurate method for the diagnosis of liver metastasis. The advantage of this approach is that the scanning section is fixed, and can be dynamically compared during the observation of the lesions. In addition, it is more objective and sensitive than ultrasound. However, the disadvantage of this method is that the specificity and sensitivity to diffuse small nodules and small cancerous lesions are poor. Hence, several cases may be misdiagnosed. Traditional CT scan and magnetic resonance imaging (MRI) cannot diagnose lung cancer in the early stage, owing to the absence of the corresponding clinical symptoms and lesions. This also hinders the early detection of liver metastasis. Therefore, the discovery of factors affecting metastasis is of great importance for the early treatment of lung cancer metastasis to the liver. Markers in the serum are widely used for the screening of clinical tumors. The detection of tumor markers in human blood, body fluids, or tissue cells can assist in determining the presence, pathogenesis, and prognosis of tumors.

Currently, numerous tumor markers in the serum have been associated with lung cancer. Mal (Ma et al., 2015) found that the levels of CEA, CA-125, and Cyfra21-1 are high in lung cancer, and can be used for the diagnosis of lung cancer. Moreover, previous studies have shown that CEA, Cyfra21-1, and CA-125 are associated with poor prognosis in non-small cell lung cancer (Cedrés et al., 2011). In addition, tumor markers may be potentially effective in predicting metastasis. However, currently, there is no evidence showing the difference between liver metastasis and non-liver metastasis of lung cancer. Therefore, in this study, the medical records of elderly patients with lung cancer from the First Affiliated Hospital of Nanchang University (Nanchang, China) were reviewed. We screened for liver metastasis based on the serological examination of a large number of elderly patients with lung cancer. The content of tumor markers in elderly patients was also compared to study the correlation between risk factors. Furthermore, we attempted to establish a standard for distinguishing between liver metastasis and non-liver metastases of lung cancer, and targeted anticancer treatment strategies for elderly patients with this disease.

The reason for choosing these biomarkers is that there have been related studies that have shown that these biomarkers are related to liver metastasis of lung cancer or other tumors, but there is no clear level indicator that when a certain biomarker exceeds how much, lung cancer may be suspected Liver metastases occur. Therefore, we can select these indicators and hope to obtain relevant biomarker levels through big data research.

## Materials and methods

### Study design

In this study, the clinical data of 3,046 elderly patients with lung cancer (188 and 2,858 elderly patients with liver and non-liver metastases, respectively) were selected in the First Affiliated Hospital of Nanchang University (Nanchang, China) from September 1999 to July 2020. Elderly patients with liver metastasis were screened, and their medical records and serological data were compared with those without liver metastasis. All elderly patients volunteered to participate in this study. The study was approved by the Medical Research Ethics Committee of the First Affiliated Hospital of Nanchang University, Nanchang, China. A pathological section, obtained through surgical resection or biopsy, was used to clearly diagnose lung cancer. Liver metastasis of lung cancer was diagnosed by CT and MRI, and data related to tumor markers in the serum were recorded.

### Data collection

We collected various clinical data from the medical records of elderly patients with liver metastasis of lung cancer, including age, gender, time of diagnosis, lesion metastasis, and treatment. The examined tumor markers in the serum included ALP, HB, AFP, CEA, NSE, Cyfra21-1, CA-125, CA-153, CA-199, and free PSA.

### Statistical analyses

We analyzed the differences between tumor markers in the liver metastasis group and the non-liver metastases group using an independent *t*-test. A binary logistic regression model was subsequently applied to identify independent risk factors of liver metastasis. We constructed a receiver operating characteristic (ROC) curve, and calculated the area under the curve (AUC). Subsequently, we calculated the cut-off value, sensitivity, and specificity of risk factors. *p* < 0.05 indicated statistical significance. All statistical analyses were performed using the SPSS 20.0 software (SPSS, IBM, United States) and Excel 2010 software (Excel, Microsoft, United States).

## Results

### Demographics and characteristics of elderly patients

**FIGURE 1:**
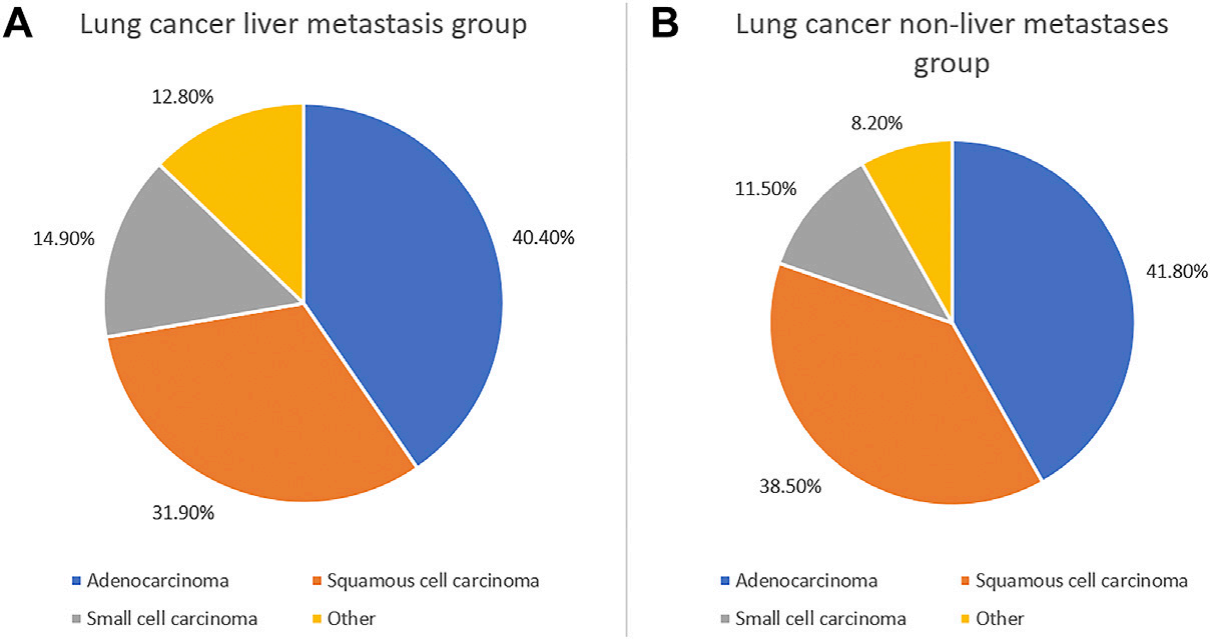
Clinical characteristics of elderly lung cancer patients with liver metastasis and without liver metastasis. The clinical characteristics of elderly patients with lung cancer liver metastasis **(A)** and without liver metastasis **(B)**.

**FIGURE 2:**
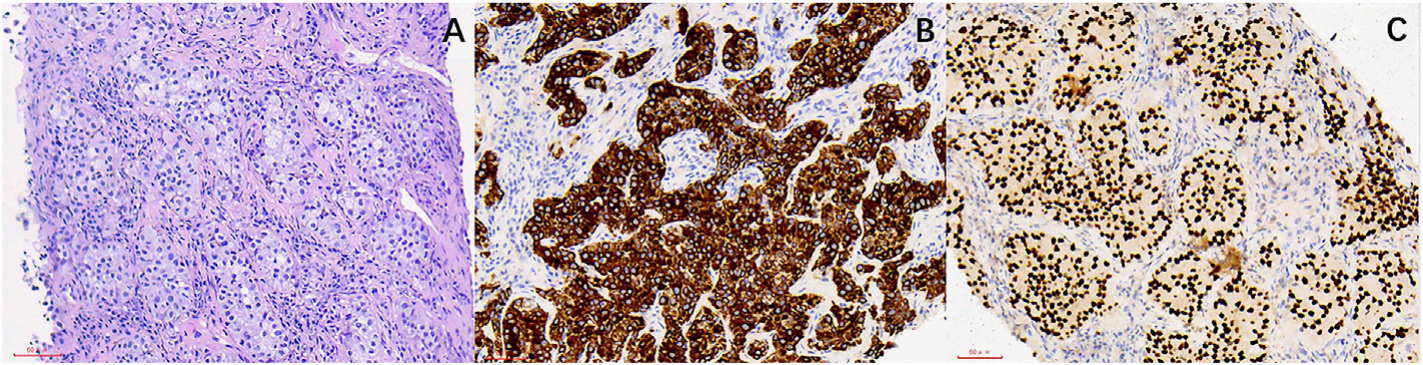
The HE staining and IHC images from lung cancer patients with liver metastasis. **(A)** Lung cancer (HE ×200). **(B)** CK7(+) (SP ×200). **(C)** TTF-1 (+) (SP ×200).

In this study, 188 cases of liver metastasis from lung cancer and 2,858 cases of lung cancer non-liver metastases were collected. The mean ages in the lung cancer liver metastasis and non-liver metastases from lung groups were 60.3 ± 4.9 and 60.6 ± 3.2 years, respectively. According to the chi-squared test and Student’s t-test, there was no significant difference between the groups in terms of gender and age (*p* > 0.05). In addition, the statistical analysis showed differences in the histopathological types between the two groups (*p* < 0.05). There was a statistically significant difference in the pathological type ratio between the liver metastasis group and non-liver metastases group (*p* = 0.003), and the proportion of adenocarcinoma was the highest. The expression of small cell carcinoma in the liver metastasis group was higher than that observed in the non-liver metastases group. The majority of elderly patients had received chemotherapy since the onset of the disease (Table 1; Figure 1). More detailed clinical data of the patients are provided in Figure 2.

**TABLE 1:** Clinical data of liver metastasis of lung cancer and elderly patients with lung cancer non-liver metastases.

Patient characteristics	Lung cancer liver metastasis group (%) (*n* = 188)	Lung cancer non-liver metastasis group (%) (*n* = 2,858)	*p* Value
Gender			
Male	124 (66.0)	1,892 (66.2)	0.214
Female	64 (34.0)	966 (33.8)	—
Age			
Mean	66.3 ± 4.9	64.6 ± 3.2	0.220
Histopathological type			
Adenocarcinoma	76 (40.4)	1,195 (41.8)	0.001
Squamous cell carcinoma	60 (31.9)	1,100 (38.5)	—
Small cell carcinoma	28 (14.9)	329 (11.5)	—
Other	24 (12.8)	234 (8.2)	—
Smoking history	101 (53.1)	1,495 (48.9)	0.253
Other transfers	131 (69.7)	538 (18.8)	—
Eyes	17 (9.0)	45 (1.5)	—
Brain	35 (18.6)	216 (7.5)	—
Bone	23 (12.2)	250 (8.7)	—

*p* < 0.05 is statistically significant.

### Clinical data and risk factors for liver metastasis of lung cancer

After comparing the data for tumor biomarkers in elderly patients with lung cancer liver metastasis and lung cancer non-liver metastases, we found that the levels of ALP, AFP, CEA, CA-125, CA-199, CA-153, Cyfra21-1, and NSE were extremely high compared with those measured in elderly patients lung cancer non-liver metastases. In contrast, the level of HB was lower in the former group (*p* < 0.05). There were no significant differences between the two groups in the levels of calcium, CA-724, and total PSA in the serum (*p* > 0.05) (Table 2). Through binary logistic regression, CA-125, Cyfra21-1, and NSE were identified as independent risk factors for the liver metastasis of lung cancer (Table 3).

**TABLE 2:** Differences in tumor biomarkers between liver metastasis of elderly lung cancer and non-liver metastases of elderly lung cancer.

Tumor biomarkers	Lung cancer liver metastasis group	Lung cancer non-liver metastasis group	t	P
ALP (U/L)	150.30 ± 11.3	89.37 ± 1.34	10.25	<0.001
Calcium (mmol/L)	2,023 ± 0.02	2.37 ± 0.09	0.41	0.068
AFP (ng/ml)	3.22 ± 0.39	1.72 ± 0.03	7.85	<0.001
CEA (ng/ml)	100.5 ± 16.57	44.07 ± 4.63	3.04	0.0024
CA-125 (U/ml)	171.2 ± 27.59	65.30 ± 3.12	7.53	<0.001
CA-199 (U/ml)	148.7 ± 51.37	40.15 ± 7.37	3.43	0.0006
CA-153 (U/ml)	33.98 ± 5.51	19.72 ± 0.96	5.48	<0.001
CA-724 (U/ml)	16.60 ± 7.52	8.30 ± 4.13	0.51	0.55
CYFRA21-1 (ng/ml)	16.07 ± 2.83	9.43 ± 0.55	2.92	0.0035
TPSA (ng/L)	1.83 ± 0.14	1.66 ± 0.07	0.58	0.5630
NSE (μg/L)	41.77 ± 5.49	24.85 ± 0.74	6.97	<0.001
HB (g/L)	112.1 ± 1.52	119.5 ± 0.36	5.15	<0.001

Independent sample Student’s t-test was used. *p* < 0.05 indicates statistical significance.

Abbreviationalkaline phosphatase (ALP), serum calcium, hemoglobin (HB), alpha-fetoprotein (AFP), carcinoembryonic antigen (CEA), neuron-specific enolase (NSE), cytokeratin fragment 19 (Cyfra21-1), carbohydrate antigen-125 (CA-125), carbohydrate antigen-153 (CA-153), carbohydrate antigen-199 (CA-199), carbohydrate antigen-724 (CA-724), total prostate‐specific antigen (TPSA).

**TABLE 3:** Risk factors for elderly patients with liver metastasis of lung cancer.

Factors	B	Exp(B)	Or (95% CI)	P
ALP	0.006	1.006	0.994–1.018	0.316
AFP	0.409	1.505	0.930–2.436	0.096
CEA	0.007	1.007	0.996–1.018	0.0024
CA-125	−0.032	0.969	0.908–1.033	< 0.001
CA-199	−0.001	0.999	0.961–1.039	<0.001
CA-153	0.049	1.003	0.999–1.006	<0.001
CYFRA21-1	−0.013	0.987	0.797–1.224	0.004
NSE	0.003	1.003	0.989–1.017	<0.001
HB	0.033	0.983	0.976–0.991	<0.001

Binary logistic analysis was applied. *p* < 0.05 indicates statistical significance.

AbbreviationsB, coefficient of regression; OR, odds ratio; CI, confidence interval, alkaline phosphatase (ALP), serum calcium, hemoglobin (HB), alpha-fetoprotein (AFP), carcinoembryonic antigen (CEA), neuron-specific enolase (NSE), cytokeratin fragment 19 (Cyfra21-1), carbohydrate antigen-125 (CA-125), carbohydrate antigen-153 (CA-153), carbohydrate antigen-199 (CA-199).

### Cut-off values, sensitivity, specificity, and AUC of CA-125, Cyfra21-1, and NSE for the diagnosis of lung cancer metastasis to the liver

**FIGURE 3:**
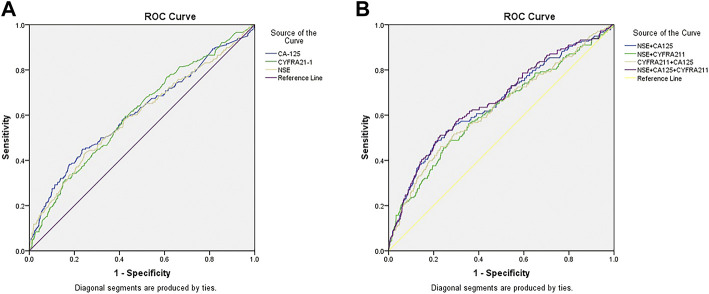
The receiver operating characteristics (ROC) curves of risk factor CA-125, CYFRA21-1, and NSE for detecting elderly lung cancer patients with liver metastasis. ROC curve of the levels of [3CA-125, CYFRA21-1, and NSE in elderly patients with liver metastasis of lung cancer **(A)**. ROC curve of the CA-125+CYFRA21-1, CA-125+NSE, CYFRA21-1+NSE combination and CA-125+CYFRA21-1+NSE combinations in elderly patients with liver metastasis of lung cancer **(B)**.

Table 4 shows that the cut-off values for CA-125, Cyfra21-1, and NSE were 53.00 U/ml, 4.15 U/ml, and 23.39 ng/ml, respectively. The AUC of Cyfra21-1 was the highest. Figure 3A shows the ROC curves for CA-125, Cyfra21-1, and NSE as a single factor. We subsequently tested the possible combinations of these three risk factors and all combinations in pairs. Figure 3B shows CA-125 + Cyfra21-1, CA-125 + NSE, Cyfra21-1+NSE, and CA-125 + Cyfra21-1+ NSE combinations. We found that the combination of CA-125 + Cyfra21-1+NSE exhibited AUC value of 0.672. The sensitivity and specificity of CA-125 + Cyfra21-1, CA-125 + NSE, Cyfra21-1+NSE, and CA-125 + Cyfra21-1+NSE can be observed in Table 4 (*p* < 0.05).

**TABLE 4:** Cut-off value, sensitivity, specificity, and AUC of CA-125, Cyfra21-1, and NSE for the diagnosis of liver metastasis in elderly patients with lung cancer.

Factor	Cut-off value	Sensitivity (%)	Specificity (%)	AUC	P
CA-125 (U/ml)	53.00	45.70	76.20	0.614	<0.001
Cyfra21-1 (U/ml)	4.15	60.10	57.10	0.616	<0.001
NSE (ng/ml)	23.39	44.10	75.00	0.608	<0.001
CA-125 + Cyfra21-1	62.36	46.80	76.70	0.631	<0.001
CA-125 + NSE	87.57	51.10	78.40	0.663	<0.001
Cyfra21-1+NSE	32.01	50.50	72.80	0.633	<0.001
CA-125 + Cyfra21-1+NSE	92.44	53.20	76.80	0.672	<0.001

Sensitivity and specificity were obtained at the point of the cut-off value. *p* < 0.05 indicates statistical significance.

AbbreviationsAUC, area under the curve; CI, confidence interval; NSE, neuron-specific enolase, Cyfra21-1, cytokeratin fragment 19, CA-125, carbohydrate antigen-125.

### Limitation

We performed serological experiments on elderly patients with liver metastases from lung cancer and those without liver metastases from lung cancer and approved the consent of the elderly patients and their families. Environmental factors may affect the experiment (for example, the contamination of individual serum by the instrument) is the limitation of the experiment. We still need more sample sizes to reduce the impact of these changes on the experimental results.

## Discussion

Lung cancer is a common malignant tumor characterized by challenging detection and lack of specificity in clinical manifestations. Most elderly patients develop distant metastasis or present with Late stage diseases such as pleural effusion, and the prognosis is poor. Therefore, early diagnosis and treatment of this disease is especially important. Tumor markers in the serum are substances that reflect the presence and growth of tumors. They mainly include proteins, polyamines, hormones, enzymes, and oncogenes. They are produced by tumor cells during tumor development or by the host. Tumor cells are released into the bloodstream. The detection of tumor markers is helpful in diagnosing tumors, evaluating treatment outcomes and prognosis, and providing guidance for treatment options. Table 5 are studies on other metastasis of lung cancer, Table 6 is the study of liver metastasis in different cancer elderly patients.

**TABLE 5:** Studies on other metastasis of lung cancer.

Author	Year	Metastasis
Ayan., et al. (Ayan et al., 2016)	2016	Bone
Roato I., et al. (Roato, 2014)	2014	Bone
Liu Y., et al. (Liu et al., 2015a)	2015	Bone
Yang F., et al. (Yang et al., 2010)	2010	Lymph node
Suzuki K., et al. (Suzuki et al., 2001)	2001	Lymph node

**TABLE 6:** Studies on the liver cancer from different cancers.

Author	Year	Diseases
Higashins K. et al. (Higashino et al., 1975)	2012	Lung cancer
Willyard C. et al. (Willyard, 2007)	2007	Lung cancer, Breast cancer, *etc.*
Marrero JA. et al. (Marrero et al., 2005)	2005	Lung cancer, Breast cancer, *etc.*

Currently, the mechanism involved in the metastasis of lung cancer has not been fully elucidated. However, it is generally thought that lung cancer tissues contain subcellular populations with different invasive and metastatic potentials. During the metastatic process, lung cancer cells complete a series of selections and sequential steps. Recent studies have shown that there are numerous similar abnormal molecules in primary tumors and brain metastases. However, these differ in terms of certain molecular changes, such as abnormalities in human epidermal growth factor receptor family receptors and expression of ligands. Although several studies have shown that CEA, CA-125, and Cyfra21-1 are prognostic factors for stage III-IV non-small cell lung cancer, (Zhang et al., 2015; Chen et al., 2018) they did not investigate the liver metastasis of lung cancer. Interestingly, the results of the present study showed that CEA and Cyfra21-1 are not independent prognostic factors for the metastasis of lung cancer to the liver. CEA was initially discovered in 1965 by Gold and Freeman. (Gold and Freedman, 1965) Since then, a large number of studies have confirmed its effectiveness as a tumor marker. However, thus far, only few studies have investigated the tumor markers in the sera of elderly patients with liver metastasis of lung cancer. Numerous studies have shown that the levels of CEA in the serum can be used as a risk factor for the prognosis of lung cancer. (Arrieta et al., 2009; Tomita et al., 2010; Grunnet and Sorensen, 2012) In addition, studies have shown that lung cancer and brain metastases in elderly patients with lower CEA concentration exhibit a better prognosis *versus* CEA-positive elderly patients. Moreover, the levels of CA-125 in the serum are independent factors affecting the prognosis of elderly patients with brain metastasis of non-small cell lung cancer. (Liu et al., 2015b) This study showed that the positive expression of CEA in the serum does not serve as a prognostic risk factor for elderly patients with liver metastasis of lung cancer. The levels of CA19-9 and CA-125 in the sera of elderly patients with liver metastasis of colorectal cancer are independent factors affecting prognosis. (Zhang et al., 2013; Sakamoto et al., 2015) However, there are few studies investigating the relationship between the levels of CA19-9 and CA-125 in the serum and prognosis of elderly patients with lung cancer. In recent years, studies have also shown that the levels of NSE in the serum are independent factors influencing the prognosis of elderly patients with lung cancer. Cyfra21-1 is a fragment of cytokeratin-19, which is present in the cytoplasm of monolayer and stratified epithelial tumor cells. It exhibits high levels in epithelial-derived tumor tissues, and can be used for the diagnosis of non-small cell lung cancer and lung squamous cell carcinoma with high specificity. (Zheng et al., 2014) CEA is one of the earliest markers for the diagnosis of lung cancer, and is mainly present in the epithelial tissue of the fetal digestive tract, pancreas, and liver. Under normal circumstances, the levels of CEA in the serum are low. However, CEA levels are high in elderly patients with gastrointestinal malignant tumors, breast cancer, lung cancer, *etc.* Different pathological types have different sensitivities. NSE is a glycolytic enzyme found in neurons and neuroendocrine tissues. It is a marker for the diagnosis of small cell lung cancer and neuroblastoma, and its levels are used to evaluate the diagnosis, treatment effect, prognosis and clinical stage of small cell carcinoma. CA-125 is a carbohydrate antigen used to diagnose epithelial ovarian cancer and endometrial cancer. It is a broad-spectrum tumor marker, especially in the diagnosis of lung adenocarcinoma. In this study, the accuracy, sensitivity, and negative predictive value of the combined detection of tumor markers in the serum were significantly higher than those observed with single markers. It is worth noting that serum tumor markers can be clinically diagnosed in lung cancer, although there is no significant difference in specificity and positive predictive value. The combined detection of tumor markers has a high diagnostic value, and can improve the sensitivity and accuracy of diagnosis. The reason for this may be that different tumor markers exhibit varied sensitivities to the detection of lung cancer, whereas single-marker detection cannot provide high sensitivity. Moreover, the combined detection of different markers may offer complementary advantages, and promote the sensitivity and accuracy of diagnosis.

However, few studies have comprehensively analyzed the distribution of liver metastasis in lung cancer. In the present study, we examined the levels of ALP, calcium, HB, AFP, CEA, CA-125, CA-199, CA-153, CA-724, Cyfra21-1, total PSA, and NSE in the sera of elderly patients The concentrations of ALP, AFP, CEA, CA-125, CA-199, CA-153, Cyfra21-1, and NSE were found to be extremely high in elderly patients with liver metastasis of lung cancer. Notably, the levels of HB were lower in elderly patients with lung cancer non-liver metastases (*p* < 0.05). AFP (Gold and Freedman, 1965; Zhang et al., 2015) has been associated with the development of liver cancer. In addition, it has also been associated with the concentration of CEA in a rat animal model. (Zhang et al., 2011) CA-199 was originally found to be expressed in the pancreas and bile ducts, and has been used as a risk factor for the diagnosis of liver metastasis of advanced pancreatic cancer. (Dong et al., 2017) Clinically, small cell lung cancer (Qu et al., 2019) can be diagnosed according to the levels of Cyfra21-1. Therefore, based on the data analyses of previous studies, we selected CEA, CA-125, and Cyfra21-1 (*p* < 0.01, *p* < 0.01, and *p* = 0.03, respectively) as independent risk factors of liver metastasis in elderly patients with lung cancer. In addition, by determining the levels of CA-125, CEA, and Cyfra21-1 (including the cut-off values, sensitivity, specificity, and AUC), we concluded that these are specific risk factors of lung cancer metastasis.

Using the final ROC curves of these serum biomarkers, we demonstrated that the cut-off values for CA-125, Cyfra21-1 and NSE were 53.00 U/ml, 4.15 U/ml, and 23.39 ng/ml, respectively. NSE concentration 53.00 U/ml is the key point for liver metastasis in elderly patients with lung cancer. Cyfra21-1 yielded the largest AUC, showing the highest accuracy in distinguishing elderly patients with lung cancer. On this basis, we conducted further detailed diagnostic analysis for liver metastasis, without providing evidence for follow-up treatment. Unlike previous studies, this study showed that the best diagnostic values for CA-125 + Cyfra21-1, CA-125 + NSE, Cyfra21-1+NSE, and CA-125 + Cyfra21-1+NSE combinations were 62.36, 87.57, 32.01, and 92.44 U/ml, respectively. We observed that the combination of CA-125 + Cyfra21-1+NSE exhibited the highest AUC value of 0.672. The CA-125 + NSE group showed high specificity. In other words, the higher levels of CA-125 and NSE are more likely to be observed in liver metastasis of lung cancer. Therefore, we suggest that the combination of CA-125 with NSE may be a useful risk factor for the prediction of liver metastasis of lung cancer.

In summary, high expression of CA-125, Cyfra21-1, and NSE in the serum may be associated with liver metastasis of lung cancer. In addition, the combination of CA-125 + Cyfra21-1+NSE may assist in the diagnosis of liver metastasis of lung cancer. The positive expression of CA-125 and NSE in the serum is a factor affecting the prognosis of elderly patients with liver metastasis of lung cancer.

## Data Availability

The datasets presented in this study can be found in online repositories. The names of the repository/repositories and accession number(s) can be found in the article/Supplementary Material.
